# Supplementary material for: Cyanobacteria Secondary Metabolites as Biotechnological Ingredients in Natural Anti-Aging Cosmetics: Potential to Overcome Hyperpigmentation, Loss of Skin Density and UV Radiation-Deleterious Effects
Source: Mar Drugs. 2022 Mar 1;20(3):183. doi: 10.3390/md20030183 (PMC8950663; doi:10.3390/md20030183)
Supplement: Supplementary file 1 [file marinedrugs-20-00183-s001.zip › marinedrugs-1608944-SI.pdf]

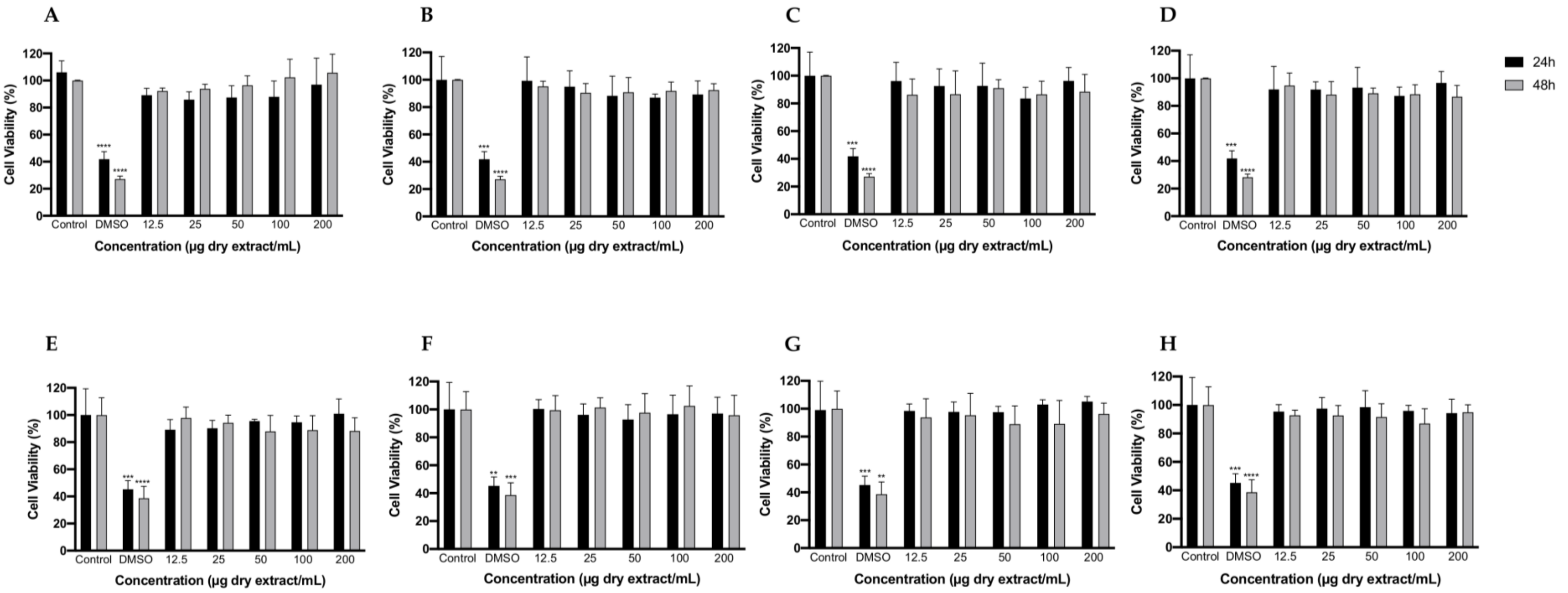

Figure 1. Keratinocyte (HaCAT) viability after 24 and 48 h of incubation with cyanobacteria aqueous (**A**–**D**) and acetone (**E**–**H**) extracts. (**A**,**E**) *Cephalothrix lacustris* LEGE 15493, (**B**,**F**) *Nodosilinea nodulosa* LEGE 06104, (**C**,**G**) *Leptolyngbya* cf. *ectocarpi* LEGE 11479, and (**D**,**H**) *Leptolyngbya boryana* LEGE 15486. Results are expressed as the percentage of MTT reduction vs. the untreated control. DMSO (20%) represents the positive control. Results are expressed as the mean ± SD of at least three independent assays, performed in quadruplicate. Statistical differences at * $p < 0.05$, ** $p < 0.01$, *** $p < 0.001$, **** $p < 0.0001$ (One way ANOVA, Tuckey HSD multiple comparisons test).

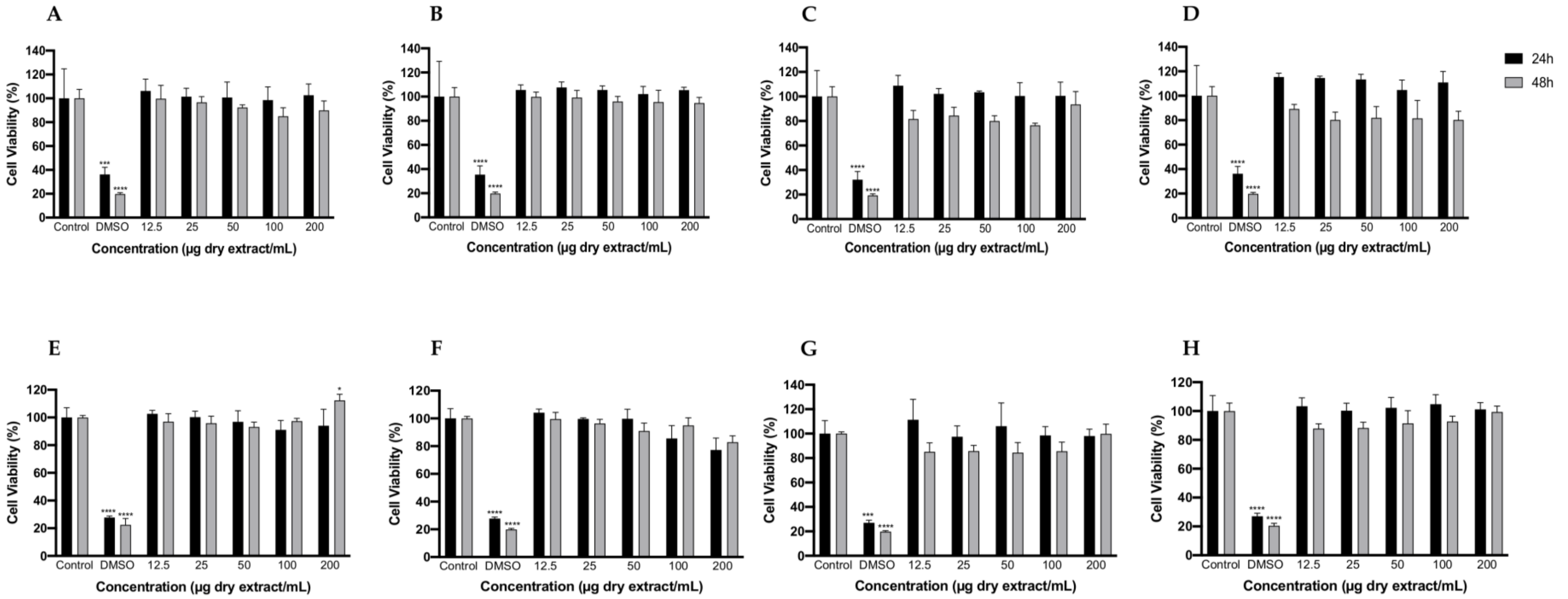

Figure 2. Fibroblast (3T3L1) viability after 24 and 48 h of incubation with cyanobacteria aqueous (**A**–**D**) and acetone (**E**–**H**) extracts. (**A**,**E**) *Cephalothrix lacustris* LEGE 15493, (**B**,**F**) *Nodosilinea nodulosa* LEGE 06104, (**C**,**G**) *Leptolyngbya* cf. *ectocarpi* LEGE 11479, and (**D**,**H**) *Leptolyngbya boryana* LEGE 15486. Results are expressed as the percentage of MTT reduction vs. the untreated control. DMSO (20%) represents the positive control. Results are expressed as the mean ± SD of at least three independent assays, performed in quadruplicate. Statistical differences at * $p < 0.05$, ** $p < 0.01$, *** $p < 0.001$, **** $p < 0.0001$ (One way ANOVA, Tuckey HSD multiple comparisons test).

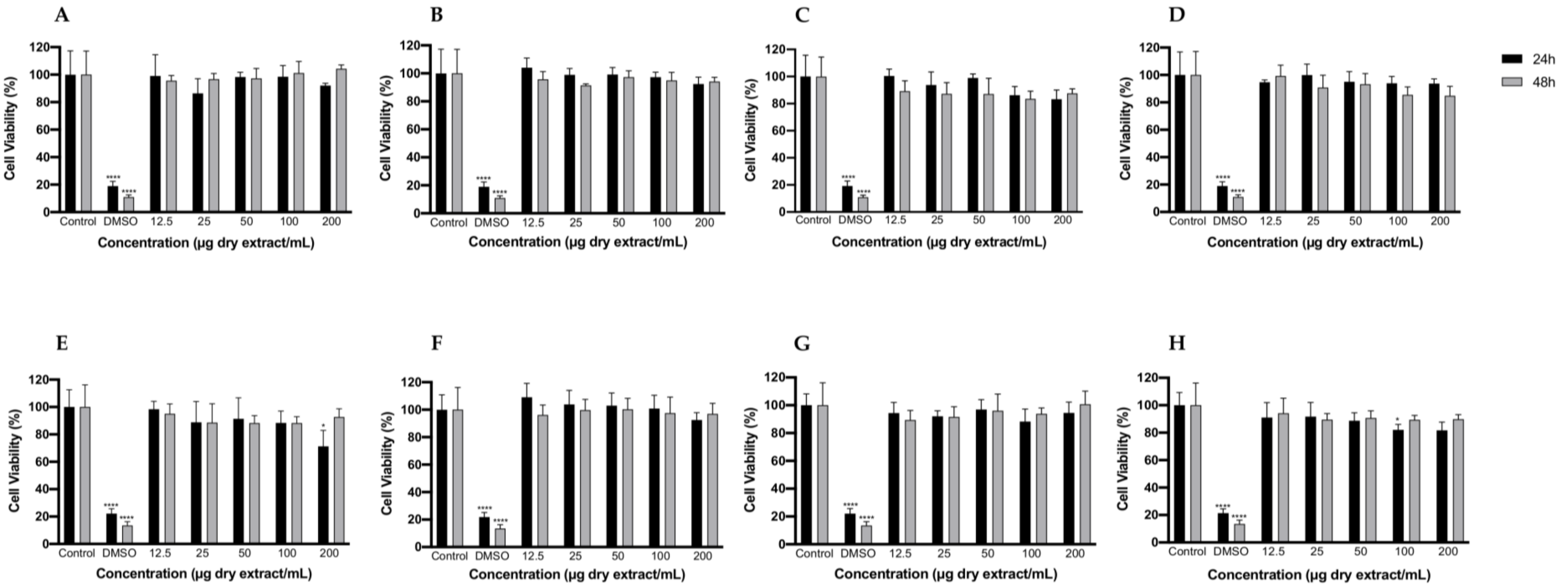

Figure 3. Endothelial cell (hCMEC) viability after 24 and 48 h of incubation with cyanobacteria aqueous (**A**–**D**) and acetone (**E**–**H**) extracts. (**A**,**E**) *Cephalothrix lacustris* LEGE 15493, (**B**,**F**) *Nodosilinea nodulosa* LEGE 06104, (**C**,**G**) *Leptolyngbya* cf. *ectocarpi* LEGE 11479, and (**D**,**H**) *Leptolyngbya boryana* LEGE 15486. Results are expressed as a percentage of MTT reduction vs. the untreated control. DMSO (20%) represents the positive control. Results are expressed as the mean ± SD of at least three independent assays, performed in quadruplicate. Statistical differences at * $p < 0.05$, ** $p < 0.01$, *** $p < 0.001$, **** $p < 0.0001$ (one way ANOVA, Tuckey HSD multiple comparisons test).
